# Prevalence of *Staphylococcus aureus* methicillin-sensitive and methicillin-resistant nasal carriage in food handlers in Lebanon: a potential source of transmission of virulent strains in the community

**DOI:** 10.1099/acmi.0.000043

**Published:** 2019-08-19

**Authors:** Marwan Osman, Khadija Kamal-Dine, Khaled El Omari, Rayane Rafei, Fouad Dabboussi, Monzer Hamze

**Affiliations:** ^1^ Laboratoire Microbiologie Santé et Environnement (LMSE), Doctoral School of Sciences and Technology, Faculty of Public Health, Lebanese University, Tripoli, Lebanon; ^2^ Quality Control Center Laboratories at the Chamber of Commerce, Industry & Agriculture of Tripoli & North Lebanon, Tripoli, Lebanon

**Keywords:** *Staphylococcus aureus*, methicillin-resistant *Staphylococcus aureus*, enterotoxin producing *Staphylococcus aureus*, food handlers, epidemiology, food poisoning, Lebanon

## Abstract

**Purpose:**

To determine the prevalence and virulence determinant genes of nasal colonization by *Staphylococcus aureus* among food handlers in Tripoli, Lebanon.

**Methodology:**

Within a cross-sectional study design, nasal swab specimens were collected. Epidemiological and microbiological investigations were performed through conventional culture and MALDI-TOF-MS. Antibiotic susceptibility patterns and genetic virulence determinants including enterotoxin genes were also investigated for all isolates.

**Results:**

The data herein show that *S. aureus* nasal carriage is highly prevalent (23.8 %), and that the rate of methicillin-resistant *S. aureus* (MRSA) carriage was twice as high as in our last report in 2008. Several enterotoxin genes were detected in five isolates including one MRSA and four methicillin-sensitive *S. aureus *.

**Conclusion:**

To our knowledge, this is the first investigation in the last decade to examine the carriage prevalence of *S. aureus* among food handlers in Lebanon. This work reports a concerning level of MRSA, and enterotoxin-producing *S. aureus* nasal carriage, which could potentially act as a contamination reservoir and lead to food poisoning.

## Full Text

*Staphylococcus aureus* is a commensal bacterium that typically colonizes the anterior nares and causes a broad spectrum of infectious diseases including staphylococcal food-borne diseases (SFD) [1]. SFD are very common worldwide and are mainly associated with the contamination of food by preformed *S. aureus* enterotoxins [2]. These are a superfamily of secreted virulence factors with potent superantigenic activity that commonly cause toxic shock-like syndromes and have been implicated in SFD [3]. Hence, the best way to prohibit the emergence of SFD is to prevent the presence of nasal colonization and hand contamination of food handlers with enterotoxin-producing *S. aureus *, and/or to keep food at a safe temperature. Eradication of *S. aureus* nasal carriage using antibiotics is effective in reducing the probability of SFD [4]. On the other hand, antimicrobial resistance (AMR) has become a global issue that continues to grow and threatens the effective treatment of infectious diseases. In Lebanon, *S. aureus* infections and its resistant forms were largely investigated in clinical settings showing high levels of AMR among isolates [5, 6]. Unfortunately, the epidemiology of and risk factors for *S. aureus* carriage in Lebanese non-hospital settings were poorly studied, particularly in the food sector. Despite the importance of this sector to the economy and industry, food safety is still a major issue in Lebanon. In this context and in order to provide insight into the epidemiology of *S. aureus* carriage in community settings in Lebanon, the main purposes of the present cross-sectional study were to screen for *S. aureus* nasal carriage, to describe the prevalence of methicillin-resistant *S. aureus* (MRSA), to determine their resistance patterns, and to detect the presence of exfoliative toxins A (*eta*) and B (*etb*), enterotoxins (*se*) and toxic shock syndrome toxin-1 (*tsst-1*) encoding genes, in Lebanese food handlers working in three major pastries in Tripoli, North Lebanon.

The study was approved by the research ethics committee of the Doctoral School of Science and Technology/Lebanese University (CE-EDST-3–2018). All participants gave written informed consent, and all data were analysed anonymously. A total of 160 individuals (ranging in age from 18 to 61 years) including 111 (69.4 %) males and 49 (30.6 %) females participated in the study during the period from June to September 2018. Out of them, 63 (39.4 %) took antibiotic treatment during the last 3 months including 26 (41.3 %) without clinical prescription.

To screen for *S. aureus *, nasal swabs were collected from the anterior nares of individuals and placed in bacterial transport medium (Yancheng-Huida, China). The samples were cultured in mannitol salt agar (Chapman, Bio-Rad, France) and incubated for 24 h at 36±1 °C. Colonies suggestive of *S. aureus* were identified using MALDI-TOF-MS (Vitek MS, bioMerieux, France). The antibiotic susceptibility testing for *S. aureus* isolates was performed by the disk diffusion method according to the EUCAST-2018 recommendations. The strains of *S. aureus,* which were found to be resistant to cefoxitin (diameter <22 mm) were identified as MRSA. Genomic DNA was extracted from isolates using the boiling method for 5 min. The detection of *eta*, *etb*, *se* (A, B, C, D and E) and *tsst-1* encoding genes was carried out as described previously [7].

Overall, a total of 38 (23.8 %) non-duplicate strains of *S. aureus* were isolated in our study. This prevalence is relatively high, but it is lower than that previously reported in a similar population in Lebanon (39 % in 2008) [8]. Moreover, other earlier studies in the Middle Eastern region documented a carriage rate of *S. aureus* among food handlers around 53 % in Kuwait [9] and 79 % in Turkey [10]. Nevertheless, the prevalence of *S. aureus* observed in our population is in the range of those reported in other epidemiological studies showing a similar prevalence such as 5.1 % in China [11], 19.8 % in Portugal [12], 22.1 and 29.3 % in Brazil [13, 14], 30.1 % in Iraq [15], 34.6 % in the USA [16], 35.7 % in Turkey [17] and 8.7–38.4 % in Lebanon [18, 19]. Hence, nasal *S. aureus* carriage rates show a large variation among investigations, locations, seasonality, age and countries [20]. Moreover, recent studies of the sensitivity of *S. aureus* carriage detection at different anatomic sites reported that only 70 % of cases were detected using nasal swabs [18]. Therefore, by extrapolation, in our investigation, we might have missed approximately 10 % of carriers.

**Fig. 1.**
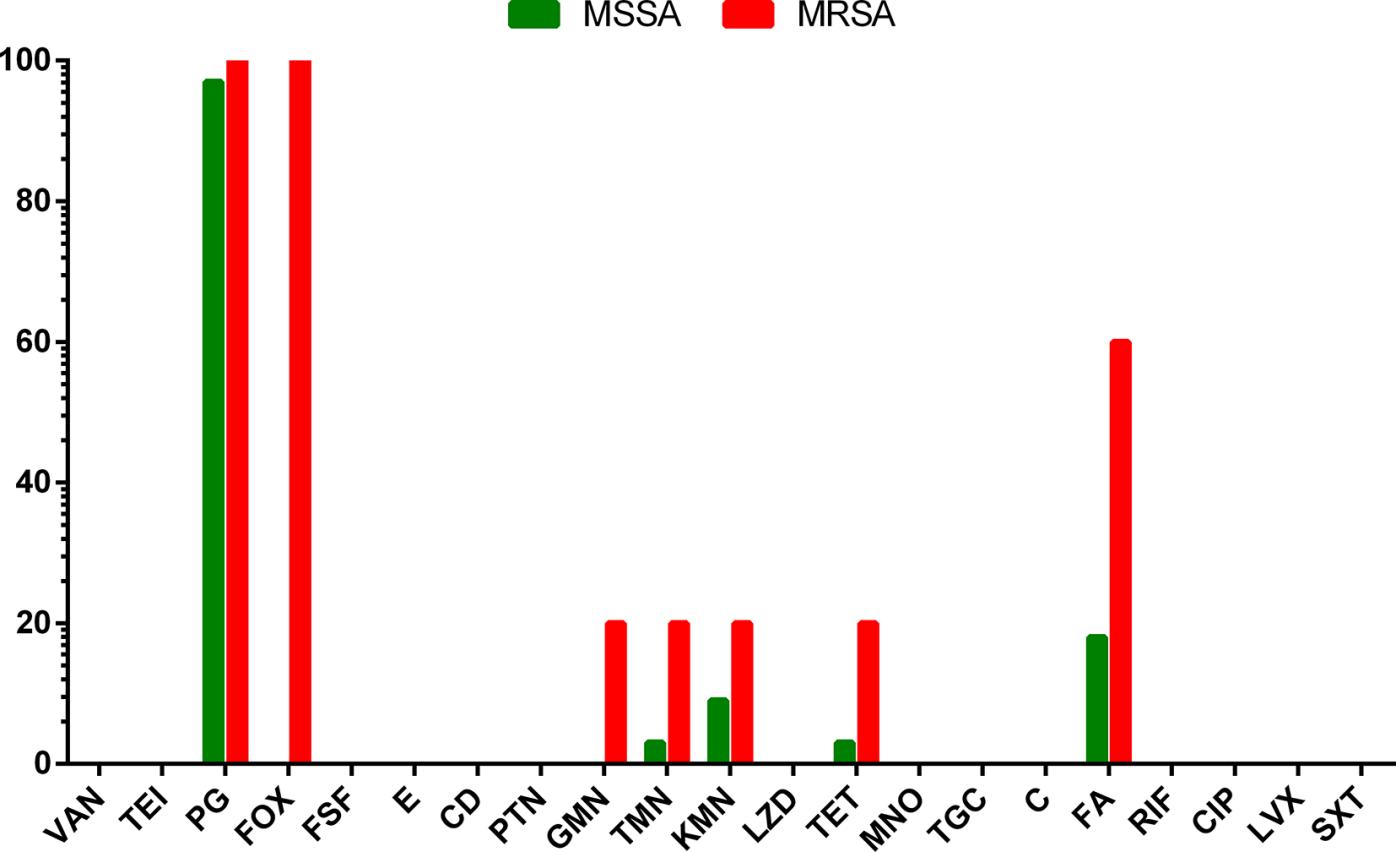
Antibiotic resistance rates of *S. aureus* isolates: comparative resistance of methicillin-sensitive *S. aureus* (MSSA) and MRSA. * VAN, vancomycin; TEI, teicoplanin; PG, penicillin G; FOX, cefoxitin; FSF, fosfomycin; E, erythromycin; CD, clindamycin; PTN, pristinamycin; GMN, gentamicin; TMN, tobramycin; KMN, kanamycin; LZD, linezolid; TET, tetracycline; MNO, minocycline; TGC, tigecycline; C, chloramphenicol; FA, fusidic acid; RIF, rifampicin; CIP, ciprofloxacin; LVX, levofloxacin; SXT, trimethoprim/sulfamethoxazole.

AMR profile of *S. aureus* isolates has been investigated and summarized in Fig. 1. Out of five isolated MRSA (13.2%), one isolate is found to be resistant to critically important antibiotics including *β*-lactams, aminoglycosides, tetracycline and fusidic acid. Although the level of MRSA is lower than that observed in clinical settings [21, 22], our data reported an alarming increase in the prevalence of MRSA in healthy carriers compared to the burden of MRSA in the last decade [8]. Our findings are higher than those reported previously among food handlers in developing countries. Numerous earlier studies showed a low level of nasal carriage rate of MRSA among food handlers with 0–5.3 % in Turkey [17, 23], 0.5 % in Kuwait [9], 0.96 % in PR China [11], 6.5 % in Lebanon [8] and 9.8 % in Ethiopia [24]. In contrast, in another study, the nasal carriage rate of MRSA was reported as 28.6 % among food handlers working in public hospitals in Brazil [14]. Regrettably, AMR becomes a major global health threat especially in developing countries and is spreading rapidly [25]. The relatively high level of MRSA nasal carriage in healthy adults working in non-hospital settings is probably reflecting the overuse and misuse of antibiotics in both the human and animal sectors in Lebanon [26–29].

In the second part of our study, we looked for virulence genes in *S. aureus* isolates. The *etb* gene was present in one methicillin-sensitive *S. aureus* (MSSA) isolate. Moreover, the *se* gene was detected in five isolates including four MSSA and one MRSA. *sea*, *sec* and *sed* genes were present in four (10.5 %), one (2.6 %) and one (2.6 %) isolates, respectively. Interestingly, one MSSA isolate harboured both *sea* and *sed* genes. However, the genes for *eta* and *tsst-1* were not detected in any isolates. *S. aureus* is the main cause of food poisoning because of its ability to produce enterotoxins, which if ingested in sufficient amounts result in sickness [9]. This study has demonstrated a low prevalence in the carriage of virulence genes in *S. aureus* isolated from healthy adults. Other Lebanese studies conducted in clinical settings reported a higher prevalence of enterotoxigenic *S. aureus *, with a predominance of *sei* gene [30]. We suggest that the low percentage of isolates harbouring toxins is probably due to the fact the population under study is healthy, and that other toxins were not targeted due to logistic reasons. However, although the number of isolates harbouring toxin genes are low, our findings support that food handlers are a potential cause of food contamination that may lead to SFD. Although equipment and the environment are common sources of food contamination in food poisoning outbreaks, food handlers are usually the main source of contamination with *S. aureus* [12]. Thus, eradication of *S. aureus* nasal carriage amongst food handlers may be beneficial to reduce the risk of SFD, particularly in the case of enterotoxin-producing strains. The study presented here has a few limitations. Due to the limited number of participants recruited to this study, the epidemiologic significance of these results remains to be confirmed. Moreover, the cross-sectional design of the study with a single nasal culture does not allow the classification of participants as persistent or intermittent carriers. Finally, due to logistic reasons, we did not attempt to identify the *S. aureus* clones circulating in North Lebanon. Molecular typing is crucial to better understand the local epidemiology of *S. aureus* and, thus, to promote the development of prevention and control strategies.

To our knowledge, this study is the first investigation in the last decade to examine the carriage prevalence of *S. aureus* among food handlers in Lebanon. Although the results of this study are based on a single nasal culture, the carriage prevalence of *S. aureus *, and even MRSA and enterotoxin-producing *S. aureus *, among participants is alarming. For a better understanding of the epidemiology of this bacteria and its transmission risk factors, further prospective cohort studies including a representative number of participants are needed. Moreover, characterization of isolates by a variety of molecular tools including staphylococcal chromosomal cassette of MRSA, spa typing, multilocus sequence typing and pulsed-field gel electrophoresis would enable epidemiological studies and should be pursued.
